# Integrative Treatment Approaches with Mind–Body Therapies in the Management of Atopic Dermatitis

**DOI:** 10.3390/jcm13185368

**Published:** 2024-09-11

**Authors:** Gil Yosipovitch, Ludivine Canchy, Bárbara Roque Ferreira, Claudia C. Aguirre, Therdpong Tempark, Roberto Takaoka, Martin Steinhoff, Laurent Misery

**Affiliations:** 1 Miami Itch Center, University of Miami, Miami, FL 33130, USA; 2 La Roche-Posay Laboratoire Dermatologique, 92300 Levallois-Perret, France; 3 Laboratoire Interactions Epithéliums Neurones (LIEN), University of Brest, 29200 Brest, France; barbara.roqueferreira@gmail.com (B.R.F.); laurent.misery@chu-brest.fr (L.M.); 4 Department of Dermatology, Algarve University Hospital Centre, ULS Algarve, 8000-386 Faro, Portugal; 5 Doctorclaudia, Inc., Los Angeles, CA 90291, USA; 6 Department of Pediatrics, King Chulalongkorn Memorial Hospital, Chulalongkorn University, Bangkok 10330, Thailand; 7 Division of Dermatology, Medical School Hospital, University of Sao Paulo, Sao Paulo 05403-000, SP, Brazil; 8 Department of Dermatology, Weill Cornell Medicine, New York, NY 10065, USA; 9 Department of Medicine, Weill Cornell Medicine-Qatar, Doha 3050, Qatar; 10 College of Health and Life Sciences, Hamad-Bin Khalifa University-Qatar, Doha 5825, Qatar; 11 Department of Dermatology & Venereology, Hamad Medical Corporation, Doha 3050, Qatar; 12 Translational Research Institute, Hamad Medical Corporation, Doha 3050, Qatar; 13 Department of Dermatology, University Hospital of Brest, 29200 Brest, France

**Keywords:** atopic dermatitis, emollient ‘plus’, holistic intervention, integrative treatment, itch, skin–brain axis, pruritus, psychological stress, scratch, sleep disturbance

## Abstract

Atopic dermatitis (AD) is a chronic inflammatory skin disease with a complex pathophysiology characterized by intense pruritus, often associated with psychological stress and atopic and non-atopic comorbidities that significantly reduce quality of life. The psychological aspects of AD and the interaction between the mind and body via the skin–brain axis have led to an interest in mind–body therapies (MBT). The aim of this article is, therefore, to reinforce the importance of psychodermatological care in AD. We performed a focused literature review on holistic practices or integrative MBT in AD, including education, cognitive behavioral therapy, habit reversal, meditation, mindfulness, hypnotherapy, eye movement desensitization and reprocessing, biofeedback, progressive muscle relaxation, autonomous sensory meridian response, music therapy, massage, and touch therapy. A multidisciplinary holistic approach with MBT, in addition to conventional pharmacologic antipruritic therapies, to break the itch–scratch cycle may improve AD outcomes and psychological well-being. Although there is a paucity of rigorously designed trials, evidence shows the potential benefits of an integrative approach on pruritus, pain, psychological stress, anxiety, depressive symptoms, and sleep quality. Relaxation and various behavioral interventions, such as habit reversal therapy for replacing harmful scratching with massaging with emollient ‘plus’, may reduce the urge to scratch, while education may improve adherence to conventional therapies.

## 1. Introduction

Atopic dermatitis (AD) is a common multifactorial inflammatory skin disease characterized by intense pruritus that often becomes intractable. AD has a substantial burden on patients/caregivers and on society [1,2]. In a systematic review on the burden of AD in adults and adolescents, itch, sleep disturbance, depression, and anxiety were the most frequently reported parameters related to the clinical and humanistic burden of AD [2]. Poor quality of life (QoL) is a predominant aspect of impaired mental wellness in patients with AD [3] and is adversely affected by higher AD severity, in addition to depression, anxiety, and psychological stress [4]. AD is associated with substantial comorbidity [5], including mental health conditions in adults and children [6,7,8,9]. Psychological conditions secondary to AD may potentially cause decreased self-esteem, social anxiety, depression, and suicidal ideation [9,10]. Adult and pediatric AD patients/caregivers indicated they are aware of the impact of AD, especially severe AD, on their mental health, and improvements in mental health can also improve dermatological outcomes [11].

However, conventional treatment approaches for AD often focus on the physical skin symptoms without sufficiently addressing the psychological comorbidities [12]. Conversely, holistic practices and integrative approaches focus on healing an individual as a whole using a psychological approach with various combinations of conventional and complementary therapies. As pruritus and psychosocial distress in patients with AD can lead to mental health comorbidities, such as anxiety and depression, patients should be screened and referred appropriately [5]. For example, integrating psychological support into the management of AD through the implementation of multidisciplinary clinics may help improve mental health [13]. Recently, the AD Quality of Care Initiative highlighted good practice interventions to treat AD patients, including a multidisciplinary and holistic approach with psychological support, patient education, and shared decision making [14]. However, comprehensive multidisciplinary and holistic models of care for dermatologic disease with psychosocial comorbidity are not widely available [15]. Although it is increasingly recognized that multidisciplinary psychodermatology clinics provide a clinical benefit to patients with psychodermatological disorders [16], possible challenges to interdisciplinary collaboration in clinical practice may include scheduling difficulties in coordinating multiple specialists, time-consuming consultations, increased (short-term) costs, and increased training requirements for clinicians [15,16,17].

The psychological aspects of AD and the interaction between the mind and body via the skin–brain axis has led to an interest in mind–body therapies (MBT) as potential adjunct treatments [18]. MBT practices (such as meditation, mindfulness-based stress reduction [MBSR], hypnotherapy, guided imagery, and biofeedback) are common complementary therapies that use the mind to influence physical functions and directly affect health [19,20]. Furthermore, recent evidence indicates that ‘stress’ can induce the release of neuromediators from the brain that communicate with the adrenal gland, immune cells, and the skin itself [21]. Vice-versa, cytokines and neuromediators in inflammatory processes can enter central neuronal cells, thereby aggravating stress responses and modulating itch, pain, or sleep, for example [22]. The mechanisms of how psychological therapies may modulate neuroimmune circuits remains an important matter of debate [23].

The aim of this article was to review the evidence for incorporating mental wellness as a dimension of dermatological care using an integrative approach.

## 2. Methods

A virtual advisory board of 8 international experts was convened in January 2024 to discuss the challenges in treating subjective symptoms, such as itch, pain, stress, and sleep disturbances, in the management of AD and the potential benefits of using an integrative treatment approach of recognized, and emerging, complementary and alternative MBT. The experts were selected for their renowned expertise in their respective fields of psychodermatology, neurosciences, dermatology, and pediatrics and were representative of different countries/regions.

We performed a focused literature search of the PubMed and Scopus databases on the use of integrative treatment approaches, holistic practices or integrative MBT, or complementary therapy or alternative therapy in AD, using the following search terms: education or therapeutic patient education (TPE), cognitive behavioral therapy (CBT), habit reversal (HR), meditation, mindfulness, hypnotherapy, eye movement desensitization and re-processing (EMDR), biofeedback, progressive muscle relaxation (PMR), autonomous sensory meridian response (ASMR), music therapy, massage, and touch therapy. We concentrated on non-interventional therapies and have excluded studies on acupuncture, traditional Chinese medicine, herbal supplements, and aromatherapy, due to possible safety concerns. Of note, general definitions of the different types of intervention have previously been described [19], but details of the various integrative MBT are not always clearly reported in publications and one or more interventions may overlap, may be combined, and may be used as adjuncts to conventional treatments.

## 3. Therapeutic Needs of Atopic Dermatitis Patients

### 3.1. Itch

Itch is the cardinal and most burdensome symptom of AD, with a significant adverse effect on QoL and mental wellness. Chronic itch sensations and associated scratching behaviors lead to the itch–scratch cycle with skin barrier damage, inflammation, and the worsening of AD [24]. Chronic itch generates stress, anxiety, and fatigue, while itch can be exacerbated by psychological aspects, namely sleep disturbances, anxiety, and depression [25,26].

In a study of 1678 AD patients, 96% indicated the most important therapeutic need was to resolve itch [27]. However, conventional treatment options for AD may provide inadequate relief for itch, pain, and scratching. A combination of emollient ‘plus’ (topical emollients with vehicle-type substances and additional active, non-medicated substances that are not topical drugs [28]) with conventional treatments may help improve pruritus [29], and there has been enormous progress with new treatments for AD. However, in RCTs evaluating pharmacological treatments for skin homeostasis and itch relief, it has been observed that no single conventional pharmacological intervention completely resolves itch for all AD patients [30]. Another aspect is that efficacy on pruritus is not always correlated with efficacy on AD lesions and, while most topical and systemic therapies for AD are effective at reducing pruritus, their quantitative efficacy on pruritus varies between treatments [31].

The brain may be an important target in the treatment of itch in AD since the alteration of central pathways of itch in AD patients are fundamental for the development and maintenance of chronic itch that can lead to habitual, almost obsessional, scratching [32]. Itch-related central pathways can be targeted using non-invasive treatments, such as psychological therapies [32]. Adopting a holistic approach for the management of itch may help address not only skin and somatosensory aspects, but also the cognitive and behavioral aspects [20]. A psychodermatological approach with multidisciplinary teams for holistic MBT interventions may be needed, within the constraints of clinical practice, to address all aspects of itch.

### 3.2. Pain

Pain may be less recognized by dermatologists and treatment guidelines than itch [33]. However, around 40% to 60% of AD patients report experiencing pain in lesional and nonlesional skin, which significantly affects QoL and mental health [34,35,36]. Skin pain, especially if severe, has been associated with increased AD severity, poor sleep, and depressive symptoms [35].

Although skin pain can be related to scratching, fissures, and/or active erythematous inflammatory lesions, a significant number of patients reporting skin pain thought it was only partially related to scratching and demonstrated no excoriations [35]. Indeed, skin pain appears to be a neuropathic symptom independent from itch and the itch–scratch cycle [33,37]. Pain generally inhibits itch, but maladaptive central inhibitory processing of itch may lead to a blunting of normal pain-induced itch suppression in AD patients [38]. The high prevalence of skin pain and its unique burden suggest that it is not adequately addressed by current therapies for AD [33] and could therefore be a target for holistic interventions.

### 3.3. Stress

Atopic dermatitis is often associated with psychological stress [39,40], which may exacerbate itch, spontaneous scratching, flares, and skin lesions [41,42,43]. In interviews of 85 AD patients, 81% indicated that emotional stress aggravated their pruritus [44]. Psychological stress may interact with immunological factors (i.e., IL-4, IL-13, IL-31, TNFα, and thymic stromal lymphopoietin [TSLP]) via central mechanisms along with an impact on endocrine organs, the peripheral nervous system, and immune resident skin cells [23,45]. Psychological stress and stress hormones have also been shown to disrupt the skin barrier function [23]. In addition to exacerbating AD, mental health distress can also result in poor treatment adherence and outcomes. Patients with AD may have more depressive symptoms and more severe psychological distress compared to patients without AD (and often worse than patients with many other chronic diseases), highlighting the importance of interventions to reduce stress [46]. Options to modulate stress-induced itch include pharmacological treatments targeting brain structures associated with stress (e.g., selective serotonin and norepinephrine reuptake inhibitors) or nonpharmacologic therapies such as CBT and other psychotherapies for stress management [41].

### 3.4. Sleep Disturbances

Sleep disturbances contribute to poor QoL in AD patients. Changes in sleep influence immune cell distribution and the associated inflammatory cytokines, suggesting a bidirectional relationship between AD and sleep [47]. Nocturnal itching and the associated scratching behavior may disrupt sleep in AD patients of all ages [48], and this has been associated with anxiety and depressive symptoms [47]. The increased incidence of anxiety and depression in AD may be partially mediated through known modifiable risk factors such as sleep quality, and interventions to address sleep problems may improve mental health outcomes [7].

AD patients indicated that treatments, in addition to relieving itch, should also allow sleep improvement, psychological relaxation, and pain relief [49]. Conventional AD therapies may not adequately target all these aspects so patients may turn to complementary (used with conventional therapies) and alternative treatments (used instead of conventional therapies). Furthermore, integrative holistic therapies may be safer alternatives than some potential pharmacological treatments, for example, melatonin and cannabinoids may be associated with side effects [30].

## 4. Integrative Treatment Approaches in the Management of Atopic Dermatitis

In addition to treating the underlying causes of AD [50], a tailored integrative holistic treatment approach may help break the itch–scratch cycle to address the cognitive, emotional, and psychosocial components [51].

Although the choice of holistic therapies may differ between different countries or cultures, it has been estimated that around half of AD patients use some form of complementary or alternative medicine, possibly due to dissatisfaction with conventional treatments and the chronic recurring nature of AD [52,53,54]. Furthermore, dissatisfaction with care, poor QoL, and psychiatric morbidity are independently associated with poor treatment adherence [55].

A 2007 systematic review and meta-analysis suggested three rationales for using psychotherapy for AD patients: to reduce the intensity of perceived stress (e.g., CBT, stress management, educational programs); relaxation to reduce stress that exacerbates AD (e.g., autogenic training); and to reduce the urge to scratch (e.g., HR) [56]. Between 1986 and 2006, eight RCTs on psychological interventions to reduce stress were identified and found to have an additive effect compared to conventional treatments for improving eczema severity, itching intensity, and/or scratching in AD patients [56]. A meta-analysis of 22 studies on psychological therapies (educational interventions and complementary therapies were excluded) for skin conditions found that psychological therapies had a medium effect on the severity of AD (g = 0.40) and psychosocial outcomes (g = 0.53), and a medium-to-large effect on itch/scratch reactions (g = 0.67) [57]. A systematic review on psychological and stress reduction interventions for eczema found promising options that can improve disease trajectory and QoL [58]. However, they highlighted the lack of standardized guidance for clinicians on how, when, and to whom they should be implemented [58]. A 2022 systematic review grouped psychological therapies (psychodynamic psychotherapy, HR, and CBT) and MBT (meditation and MBSR, PMR, music resonance therapy, and various educational and multidisciplinary approaches) for various skin diseases (15 studies on AD) [51]. They recommended that these therapies, especially HR, should be considered as adjuvant treatment options for ADwith various multidisciplinary approaches [51]. The results of two similar online surveys amongst patients and physicians showed both groups prefer shared decision-making, although improvements are needed, such as the development of support tools, training of physicians, and education of patients [59].

Therapeutic patient education (TPE), CBT, and HR are the most widely accepted integrative treatment approaches in AD patients, while there is a paucity of robust, double-blinded, placebo-controlled studies in AD patients for other MBT [60]. However, MBT have been shown to be safe and potentially effective for pain and anxiety [19]. Furthermore, a US survey reported that children and adolescents were more likely to use MBT if they experienced pain-related conditions or emotional, behavioral, or mental conditions and if they received specialty or mental health care [61]. Protocols for psychological therapies for children, for example, TPE, HR, CBT, and mindfulness, should be adapted for their age and can be applied individually or in groups, but it is important to also include the parents/caregivers [62].

Below, we describe studies, especially RCT, on holistic approaches with multiple potential benefits on AD, pruritus, and pain, as well as on psychological stress, anxiety, sleep disorders, and treatment adherence to improve patients’ well-being.

### 4.1. Therapeutic Patient Education

TPE is a continuous process to train and guide the patient and their family to help manage the disease; it is a broad term, and details of interventions used in published studies are not always provided, but often consists of psychology and HR sessions. Consensus-based clinical guidelines for AD recommend patient education programs (structured atopic schools), as an adjunct to conventional therapy, for AD in children and adults [60,63]. Most importantly, these consensus guidelines also specify that effective conventional treatment should not be delayed [60,63].

Various formats and content can be adapted to a patient’s individual educational and cultural background [64,65]. Following good results in Europe, an eczema school was established in the USA with a multidisciplinary team consisting of a dermatologist, psychologist, nutritionist, and nurse practitioner to educate patients/caregivers on proper skin care, the itch–scratch cycle, healthy nutrition, and the role of stress in AD [66].

A recent systematic review found that online AD education can be as effective as in-person interventions, in both adult and pediatric populations, and reduce barriers to patient education [67]. Overall, educational interventions were shown to have a significant impact on reducing AD severity [67]. Most studies involved education about disease management, self-care techniques, avoidance of triggers, and comprehensive understanding of the disease process.

Interventions including patient education and AD action plans can improve treatment adherence (e.g., for corticosteroids) and clinical outcomes by raising awareness of poor adherence and helping patients develop better adherence habits [68]. Educational sessions may benefit patients with anxiety and those patients who are embarrassed by their disease by helping them cope with AD and by providing specific training (e.g., HR) to break the itch–scratch cycle [69,70]. In an RCT, adult patients with moderate-to-severe AD who received structured education from a comprehensive training manual showed more significant improvement than the control group for coping behavior for itch, QoL, and AD severity [69].

An analysis of data from 274 child–parent pairs found parents with previous negative treatment experiences, and poor coping strategies for scratch control, benefited the most from an education program [71]. Effective and standardized programs require well-trained educators to tailor programs to patients and their families. For example, video education of parents or caregivers of AD patients can be used to alleviate concerns about side effects of topical corticosteroids [72]. An RCT of 580 children (aged 2–14 years) in China with moderate-to-severe AD, found TPE decreased AD severity, improved QoL, and improved knowledge of emollient use [73], highlighting the importance of starting a program as soon as possible after disease onset.

For children with AD, the role of patient organizations and support groups, as well as networking and education should be highlighted, while treatment plans should incorporate holistic care [74,75]. Attending family support groups improved pruritus and QoL for AD patients and may be effective in the management of recalcitrant forms of AD [74]. A 2020 systematic meta-analysis of eight RCTs concluded health education could improve AD severity in children and improve QoL [76].

In children with AD, a multidisciplinary holistic approach, comprising systemic, topical, and psychological therapies, including education to provide information and practical guidance along with coping strategies for itching and sleep disturbance, is recommended to treat the dermatosis and associated pruritus, and improve QoL [77]. Parents play a key role in the treatment of AD in their children and TPE in a group or individually can help them to better understand the disease and provide practical guidance on using local treatment, coping strategies for itching and sleep disturbance, as well as distraction techniques [77].

### 4.2. CBT and Habit Reversal

CBT is a form of psychotherapy and may include a range of techniques such as cognitive restructuring, relaxation, and habit reversal. A systematic review of psychological therapies and MBT concluded the most promising interventions are CBT, HR, and MBSR interventions [51]. A review on self-esteem and identity found that AD has significant behavioral effects on children, which potentially have a negative effect on the ability to develop adequate coping mechanisms, thus highlighting the importance of empowerment and cognitive adaptation [78].

Results from an RCT in 32 adult patients with mild-to-severe AD suggest that 6 months of psychotherapy (focusing on illness perception, disfigurement, body image and bodily sensations, and anxiety related to itch–scratch patterns) was more likely to improve the anxiety and AD severity in those AD patients with the highest anxiety levels [79]. AD patients suffering from anxiety should be provided the tools to manage the unpredictability of their flares and/or gain more control by recognizing the triggers. Through education and HR, patients can be made aware of early signals of skin barrier damage (e.g., increased itch, erythematous lesions) and then take appropriate action (e.g., applying emollients, topical treatments, and/or using relaxation techniques). HR can be aimed at breaking the scratching habit that causes skin damage and worsens AD by replacing it with a more desirable habit to improve QoL [80]. For example, behavioral modification and massages with adapted emollients, especially emollients ‘plus’, may help AD patients to control their scratching impulse [28,50,81].

A 2022 systematic review that included 20 RCTs found psychological interventions, especially HR, showed a greater benefit on AD severity than educational interventions [82]. An RCT comparing four therapies for AD found CBT (comprising relaxation, self-control of scratching, and stress management) improved AD and reduced topical steroid use [83]. Psychological treatments led to significantly larger improvements in skin condition than intensive education or conventional treatment [83]. Therapist-guided internet-delivered mindfulness, exposure, and response prevention, a form of CBT, reduced AD symptoms and stress [84]. When used as an adjunct to corticosteroid treatment, HR improved the mean objective AD index score (Severity SCOring of AD [SCORAD]) [85]. HR itch-coping training with corticosteroids improved skin status (Eczema Area and Severity Index [EASI] score), acceptance, and health-related QoL while reducing scratching and catastrophizing [70]. After HR (with topical corticosteroids), the skin status improved and was strongly correlated with a reduction in scratching [86]. Finally, HR reversed harmful responses to itch and improved self-reported skin status [87].

### 4.3. Meditation and Mindfulness

Mindfulness is intentionally and non-judgmentally paying attention to the present moment to manage stress and improve overall well-being [88]. Many types of meditation exist, but the two most researched in the scientific literature are MBSR using meditative techniques and autogenic training (AT).

A systematic review in AD patients reported benefits of mindfulness-based interventions [58]. Mindfulness improved well-being, QoL, and stress levels in synergy with conventional treatments [89]. A recent RCT in 107 adults with moderate-to-severe AD compared integrated online MBSR and mindful self-compassion training with a waiting list control group (in addition to usual dermatological care in both groups); greater improvements in QoL (Dermatology Life Quality Index [DLQI] score), AD severity, itch visual analog scores (VAS), and scratching VAS were observed in the MBSR/self-compassion training group [90]. An Integrated Body–Mind–Spirit psychoeducational program (based on traditional Chinese philosophy) on stress management, awareness, balance, relaxation, guided imagery, and HR in children with AD improved SCORAD scores with a psychological benefit on anxiety [91]. A psychoeducational stress management program that included practicing awareness, self-monitoring, control, and relaxation in addition to standard medical therapy showed similar results [92]. Additionally, an RCT comparing four group treatments found AT for relaxation led to significantly larger improvement in AD and itch than standard treatment [83].

Interestingly, while meditation and self-compassion may improve QoL, recent population data found broader religiousness/spirituality may have a similar effect; AD patients who self-reported having high religiousness/spirituality were found to have a significantly lower prevalence of major depressive disorder and generalized anxiety disorder [93]. Finally, several RCTs have demonstrated short-term improvements from mindfulness and meditation in other inflammatory skin diseases, such as psoriasis [94].

### 4.4. Hypnotherapy

Hypnosis creates an altered state of consciousness that renders the mind vulnerable to direct suggestions. The two most studied disciplines of hypnotherapy to induce the trance-like state are guided imagery using the imagination and the visualization of images, sceneries or stories, and EMDR using eye movement.

Generally, high and medium hypnotizable subjects (people susceptible to hypnosis) respond better than low hypnotizable patients [95]. It was previously considered that hypnosis should only be undertaken by appropriately trained and regulated practitioners [96], but interest is increasing in internet-delivered hypnosis and self-hypnosis. A recent cross-sectional qualitative analysis following online clinical hypnosis training for chronic pain highlighted the importance of addressing misconceptions amongst clinicians, developing skills and adapting the sessions around current treatments [97].

A 2022 systematic meta-analysis included four studies on hypnotherapy in AD but only one RCT [58]. A prospective, uncontrolled clinical trial on an EMDR method showed promising results for scratching behavior, disease activity, QoL, and self-control [98]. An uncontrolled study on guided imagery for AD showed improvements in itching and scratching for AD patients with a significant difference in EASI scores before and after hypnosis [99].

Hypnosis should be adapted to the age of the patients. In a pilot study in patients with AD refractory to conventional treatment (such as topical steroids and emollients), the hypnosis program for adults (*n* = 18) involved relaxation, stress management, direct suggestions to prevent scratching and promote skin comfort, ego strengthening to relieve anxiety, and training in self-hypnosis with guided audio recordings [100]. In the same study, children (*n* = 20) were provided a recording of ‘magic music’ with the same aim [100]. Preliminary results showed a statistically significant benefit from hypnosis (on itch, scratch, sleep, and discomfort) for up to 2 years and an apparent, significant, immediate benefit in children [100]. In another study, children over 5 years old (*n* = 11) with moderately severe eczema were assessed by a clinical psychologist and taught self-hypnosis by guided imagery for relieving itch and improving relaxation. The mean eczema score (on a scale from 0 = absent to 3 = severe for dryness, lichenification, crusting, erythema, excoriations, and extent of body involved) improved over 18 weeks but was not significant [101].

In an RCT, the cutaneous pain threshold increased for both healthy controls and AD patients receiving hypnotherapy and was correlated with AD symptom improvement versus no change for the control group [102]. In a four-arm RCT, potential beneficial changes were observed in AD compared to the baseline after 16 weeks of hypnotherapy in perceived itching intensity, disease severity, and disease-specific QoL [103].

### 4.5. Biofeedback

Biofeedback, which measures patients’ physiological responses with visual or auditory feedback to permit conscious awareness to assist patients in learning to control the responses, can help patients to relax and thus may improve AD that flares with stress [95]. Furthermore, the effects obtained by biofeedback may be enhanced by hypnosis [104].

Case series showed mixed results of frontal electromyographic biofeedback on AD [105,106]. In an RCT, patients with dyshidrotic eczema received biofeedback training to decrease skin conductance and showed clinical improvement and decreased anxiety [107]. In another RCT, 44 children (aged 5–15 years) with inadequately controlled eczema received face-to-face guided imagery hypnotherapy (relaxation focused on reducing itching) or biofeedback about their level of relaxation (relaxation without direct imagery) to control symptoms [108]. Both the hypnotherapy group, especially the female patients, and biofeedback group showed significant reductions from baseline in lesion surface damage and lichenification (using a newly developed unvalidated severity measure) compared to the discussion-only control group [108].

### 4.6. Relaxation/Progressive Muscle Relaxation Therapy

Methods of stress reduction also involve physical relaxation (Progressive Muscle Relaxation Therapy [PMR]). Two case series concluded that the release of cervical muscle tension may improve both psychological stress, pruritus, and symptoms of moderate-to-severe AD [109,110].

An RCT in 25 children and adults with AD investigated tensing and relaxing various muscle groups (physical component of PMR), while focusing on the sensations of tension and relaxation (mental component) [111]. After one month of PMR, pruritus and sleep disturbance decreased significantly, and State-Trait Anxiety Inventory (STAI) scores showed significant improvement compared to the control group [111]. Therefore, PMR may be a useful adjunctive modality for the management of pruritus and sleep disturbances in patients with AD by reducing anxiety [111].

### 4.7. Autonomous Sensory Meridian Response/Sound Therapy

Autonomous sensory meridian response (ASMR) is a tingling, static-like, or goosebump sensation in response to specific audio or visual stimuli. These stimuli (‘ASMR triggers’) often elicit a calm and positive emotional state that may last up to several minutes and promote relaxation and sleep.

Although we found no studies on ASMR for AD, ASMR experiences may overlap with mindfulness [112].

### 4.8. Music Therapy

Music research from psychology, neuroscience, and psychiatry has recently been analyzed to propose a framework including four core elements of human musicality: tonality and applications targeting mood and anxiety; rhythm targeting mood, cognition, and motivation; reward to stimulate positive affect and normalizing brain reward function; and sociality for social connectedness [113].

Clinical music interventions (music forms used in the medical setting) may reduce depression, pain, anxiety, and stress about pruritus in AD patients, but further studies are needed [114,115].

Also, listening to certain types of music may have positive effects on the immune and inflammatory response [116,117]. In an RCT in 50 adult patients with moderate AD, listening to music reduced allergic skin wheal responses and in vitro allergen-specific IgE production in AD patients with latex allergy [116]. In another RCT in adult patients with chronic pruritus (*n* = 8 AD patients) comparing a single music session versus emollient use, anxiety decreased in both groups, while pruritus intensity decreased more significantly in the music group [118].

### 4.9. Massage Therapy/Touch

While afferent C-fiber stimulation is usually associated with pain, temperature, or itch, CT-fibers (c-tactile afferents or CTs) are stimulated optimally by a stimulus not in the nociceptor range but by a gentle, low-force stroking for the pleasurable properties of touch [119]. The overlapping CT affective touch system may counteract the signals of the afferent C-fiber system mediating itch and pain [120]. Therefore, massage therapy that may stimulate fibers for pleasant affective touch could reduce the perception of unpleasurable sensations, such as pain and itch, and elicit positive feelings to provide a complementary, non-pharmacological means of treating both the physical and psychological aspects of itch and AD [120].

In an RCT evaluating the effect of massage on AD in infants (≤12 months old), the massage group (who received instructions on routine care and on mother-performed massage) showed statistically significantly better EASI and QoL and had a lower relapse rate compared to the control group who received instructions on routine care only (application of emollient, avoidance of skin irritants, avoidance of allergens in maternal and infantile diet, and breast-feeding when possible). Also, the anxiety (SAS) and depression (SDS) 20-item self-reported assessment scores of the mothers improved in the massage group [121]. In a second RCT in children (3.8 years of mean age), massage significantly improved erythema, lichenification, excoriation, and pruritus compared to the control group [122]. Also, the parents had lower anxiety after the massage sessions [122].

Massages with adapted emollients, especially emollients ‘plus’ [28], should be considered to prevent xerosis and pruritus and thus improve QoL [50]; this could be combined with HR behavioral modification to control the scratching impulse and avoid skin barrier damage.

## 5. Overall Evidence

The main literature is summarized in Table 1. Although techniques are not always clearly defined, may overlap, and may be studied in the presence of various conventional treatments, the overall body of evidence appears to show that integrative treatment approaches may provide some benefits in the clinical management of AD.

## 6. Conclusions and Future Directions

The complex pathophysiology of AD requires comprehensive therapeutic strategies to manage multiple aspects of the disease, including skin barrier defects, inflammation mechanisms, and the itch–scratch cycle. As AD has psychophysiological aspects, it may respond well to techniques that help to counteract stress, such as biofeedback, CBT, HR, hypnosis, MBSR, or PMR. Reducing stress is important as this is known to exacerbate itch, while holistic interventions can combat stress and scratching behavior for chronic itch. Behavioral interventions may reduce the urge to scratch while replacing a harmful behavior with a beneficial one, such as massaging with an emollient. Furthermore, using a holistic approach to improve the itch–scratch behavior may also improve both skin and psychological outcomes as anxiety and mood disorders, such as depressive symptoms, that are related to the negative impact of chronic itch on QoL. Also, improving relaxation should reduce stress and anxiety, while improving mental health, sleep, and coping strategies related to the disease and associated chronic itch. Meditation, music therapy, progressive relaxation, hypnosis, and other stress-reducing methods can enhance overall health and resiliency, contributing to a better management of psychological stress and thus potentially have a positive impact on the progression of AD.

Holistic therapies as adjunct therapies to conventional pharmacologic antipruritic treatments may have a good benefit/risk ratio with MBT tailored to the individual patient’s needs and preferences depending on their age, culture, and psychological needs, along with education and joint decision-making to improve adherence and synergistically enhance the response to conventional treatments. Importantly, guidelines stress that effective conventional dermatological treatments should not be delayed when using an integrative treatment approach.

There are numerous limitations to the studies on MBT in AD, including small sample sizes, short-term studies, no blinding (often impossible), lack of details on intervention design, in the presence or absence of various conventional treatments, different or overlapping methodologies, and lack of adequate outcome measures. A further limitation is that the AD patient or their parent or caregiver may need to receive training in the respective technique, for example, self-hypnosis, meditation, or massage. Some holistic treatments may work better on some patients than others, and some interventions, such as relaxation techniques, may be difficult to evaluate in young children. Consequently, some modalities, for example, those involving reading or specific instructions for adults, should be adapted for use in children. Techniques that are particularly suitable for infants and children should be started as soon as possible after disease onset and include family support groups and education with practical guidance on coping strategies for itching and sleep disturbance. Since AD may cause behavioral problems in children, CBT and HR are also particularly useful for children and can help reduce distress related to chronic itch. Additionally, using HR therapy to replace harmful scratching with beneficial parent-performed massage may be particularly useful in infants for optimizing emollient use while helping to relieve stress in both the infant and parent/caregiver.

We found evidence from RCT studies that MBT may have a positive impact on various aspects of AD, for example, TPE decreased AD severity and improved QoL; CBT and HR studies showed benefits on AD severity, anxiety, stress and depressive symptoms, and QoL; mindfulness improved AD severity, pruritus, scratching, and QoL; hypnotherapy decreased pain; biofeedback decreased anxiety (in dyshidrotic eczema); PMR improved pruritus, sleep, and state anxiety; and massage therapy improved AD score, anxiety depression, and QoL.

Despite the paucity of large rigorously designed trials and no studies that directly compare different integrative treatment modalities, the overall body of evidence gathered in this review highlights the potential benefits of a multidisciplinary and integrative approach to improve the clinical management of AD. Further studies are warranted, especially as integrative treatment approaches are becoming more accessible with the development of online interventions that the patients/caregivers could potentially perform at home. Also, future studies to examine which specific patient populations of AD would be most susceptible to each integrative treatment approach would be timely.

## Figures and Tables

**Table 1:** Summary of therapeutic patient education and psychological treatment for atopic dermatitis patients from the literature.

**Therapeutic Patient Education**			
**Methods**	**AD** **Patients**	**Outcomes**	**Type of Study**
Eczema school/Health education	Children and their families	Improved eczema severity, QoL	- Meta-analysis of RCTs 2020 [76]
Online AD education	Adults Children and their families	Significant improvement in eczema severity and QoL in AD patients	- Systematic review and meta-analysis 2024 [67]
**Psychological Treatments**			
**Methods**	**AD** **Patients**	**Assessment**	**Type of Study**
Habit reversal therapy (HRT)	Children Adults	Significant reduction in eczema severity, itching intensity, and scratching. Improved QoL	- Systematic review and meta-analysis 2007 [56] - Systematic review 2022 [51] - Systematic review 2022 [58] - Systematic Review 2023 [82]
Cognitive–behavioral therapy (CBT)	Adults		
Mindfulness-based stress reduction (MBSR) Meditation/Self-compassion	Adults Children	Significant reduction in eczema severity, itching intensity, scratching, and anxiety. Improved QoL	- Systematic review 2022 [58] - RCT 2023 [90], RCT 2020 [91]
Hypnotherapy	Adults Children	Reductions in pain. Some benefits for itch, disease severity, and QoL	- Systematic literature review 2022 [58] - RCT 2023 [103]
Biofeedback	Children	Improved eczema severity	- RCT 1993 [108]
Relaxation techniques/Progressive muscle relaxation (PMR)	Adults Children	Reduction in pruritus, sleep disturbances, and anxiety	- Systematic literature review 2022 [58] - RCT 2012 [111]
Music therapy	Adults Children	Reduction in pruritus	- Systematic literature review 2022 [58] - RCT 2003 [116], RCT 2020 [118]
Massage therapy	Children	Reduction in eczema severity, pruritus, and anxiety	- Systematic literature review 2022 [58] - RCT 1998 [122]
Others: - Autogenic training - Brief dynamic psychotherapy - Structured educational programs	Children Adults	Significant reduction in eczema severity, itching intensity, and scratching in AD patients	- Systematic review and meta-analysis 2007 [56]

Abbreviations: AD, atopic dermatitis; QoL, quality of life; RCT, randomized controlled trial.

## Abbreviations

AD	Atopic dermatitis
ASMR	Autonomous sensory meridian response
CBT	Cognitive behavioral therapy
EASI	Eczema area and severity index
EDMR	Eye movement desensitization and reprocessing
HR	Habit reversal
IBMS	Integrated body–mind–spirit
MBSR	Mindfulness-based stress reduction
MBT	Mind–body therapy
PMR	Progressive muscle relaxation
QoL	Quality of life
RCT	Randomized controlled trials
TPE	Therapeutic patient education
